# Resource Signaling via Blood Glucose in Embodied Decision Making

**DOI:** 10.3389/fpsyg.2018.01965

**Published:** 2018-10-15

**Authors:** Xiao-Tian Wang

**Affiliations:** ^1^School of Humanities and Social Science, The Chinese University of Hong Kong, Shenzhen, Shenzen, China; ^2^Department of Psychology, University of South Dakota, Vermillion, SD, United States

**Keywords:** signaling, resource management, caloric metabolism, blood glucose, embodied cognition, decision neuroscience, delay discounting, self-control

## Abstract

Food, money, and time are exchangeable resources essential for survival and reproduction. Individuals live within finite budgets of these resources and make tradeoffs between money and time when making intertemporal choices between an immediate smaller reward and a delayed lager reward. In this paper, I examine signaling functions of blood glucose in regulating behaviors related to resource regulations beyond caloric metabolisms. These behavioral regulations include choices between energy expenditure and energy conservation, monetary intertemporal choices, and self-control in overcoming temptations. I begin by comparing potential embodied signals for resource forecasting and proactive decision making in terms of their pros and cons as a signal for regulating both metabolism and behavioral decision making and self-control. Based on this analysis, circulating glucose emerges as not only the designated fuel for brain metabolism but also a privileged resource forecasting signal for regulating immediate, short-term, and long-term behavioral adaptations to the resource budget of the decision maker. In the context of an on-going debate between the limited resource model and the motivation accounts of behavioral effects of blood glucose, I propose a dual functions (caloric provision and resource forecasting) and dual signaling (glucose taste and ingestion) hypothesis of circulating glucose in resource management, and provide behavioral and neurophysiological evidence of the separate effects of glucose taste to motivate effort for resource acquisition and glucose ingestion to promote resource conservation and future orientation. Accumulating evidence indicates that the body is able to detect fake signals of non-caloric sweeteners and react to such “caloric crisis” with an enhanced preference for immediate rewards over future rewards, revealing the wisdom of the body.

## Signaling in Resource Management

Resources for survival and reproduction exist in different forms. Among them, natural, caloric, somatic, and monetary resources are a few primary forms. Across the lifespan, humans make tradeoffs among different forms of resources (Stearns, 1992; Roff, 2002; Kaplan and Gangestad, 2005), according to their relative importance in different stages of life and for different tasks (e.g., growth, mating, and parenting). Foraging strategies are sensitive to body caloric budget. Birds take greater foraging risks when their expected energy budget is below a minimum caloric requirement and vice versa when the energy bought is above the colorific requirement (Stephens and Krebs, 1986). Similar strategies apply to human risky decision making concerning monetary requirements in modern times (Wang, 2002; Higginson et al., 2018).

In this paper, resource management includes the physiological and behavioral acquisition, allocation, expenditure, and conservation of caloric or monetary resources. The focus of the discussion is on the interactions between bodily metabolic signals and behavioral adjustments in resource management beyond metabolism. More specifically, this paper examines how caloric and metabolic signals, particularly the circulating blood glucose levels, affect behavioral decision making and self-control.

I propose a dual function (caloric provision and resource forecasting) and dual signaling (glucose taste and ingestion) hypothesis of circulating blood glucose. First, I provide a framework of honest and false signaling of body resource needs and budget. Second, I identify several candidates for embodied resource signaling, and compare the candidates in a cost-benefit analysis in terms of their feasibility and efficiency as a signal of body resource conditions. Among these candidates, circulating glucose stands out as an effective signal for indicating body energy budget. I propose that circulating glucose is an integral part of the energy budget forecasting mechanisms, which assess life-history related temporal tradeoffs of whether to expend energy in the present or whether to build up and wait to expend energy in the future. Energy budget signaling is proactive in that small trends in blood glucose levels can be detected before subsequent substantial changes in caloric conditions occur. Thus, minor fluctuations in blood glucose levels, although insufficient for caloric provision, can result in significant behavioral changes in the temporal tradeoffs between immediate smaller rewards and delayed larger rewards. From this perspective, blood glucose levels serve as anticipatory indicators of an organism’s energy budget and thus, resource that can be used to survive and reproduce into the future. I provide behavioral and neurophysiological evidence for the dual function and dual signaling hypothesis of blood glucose toward the conclusion that the human body is smart in telling apart fake from real caloric crisis and behaves accordingly and adaptively.

The studies of food restriction or deprivation clearly exhibit the power of the reinforcement value of a food reward. One of the lessons from the famous Minnesota starvation study conducted more than 60 years ago is that extreme hunger overrides many other fundamental drives for sex, attachment, and social status (Keys et al., 1950). Metabolic experience can exert effects on health and behavior though immediate physiological feedback, and short-term and long-term behavioral regulations (Benton, 2010; Berthoud, 2011; de Groot et al., 2011), resulting in even transgenerational changes via epigenetic mechanisms (De Rooij et al., 2010; Laus et al., 2011; Roseboom et al., 2011). Studies on human life-history evolution have suggested that food provisioning between generations and maintenance of daily calorie intake in a family have been driving forces that shape and bind together human nuclear and extended families (e.g., Hooper et al., 2015).

In a world of limited resources, individuals make decisions to consume or conserve caloric and monetary resources based on external cues and internal signals about resource situations. The concept of signaling holds a central position in studies of social communications between individuals (Otte, 1974) as well as physiological regulations within an organism (Berthoud, 2011). Both cues and signals are used in communication and decision making. A signal is a feature of an organism evolved to carry specific information in specific contexts to influence behavior of intended receivers. In contrast, a cue is not evolved for intended receivers, but is a feature of an organism or environment that can be used by receivers to infer a hidden quality (Hasson, 1997; Maynard Smith and Harper, 2003; Saleh et al., 2007). For embodied signals, the intended receivers are the signalers themselves.

From an evolutionary perspective, resource signaling is necessary given that a primary task of human foraging is to “proactively adapting to the perceived food insecurity” (Brunstrom and Cheon, 2018, p. 261). Proactive foraging relies on resource signaling and forecasting that can be carried out either by overt behaviors or by covert embodied changes. Resource signaling can be carried out by overt behaviors in social communications between different individuals or by covert metabolic signals in embodied regulations. Like other signals, the signals for resource management can be either honest and genuine or dishonest and fake (Zahavi et al., 1997).

**FIGURE 1:**
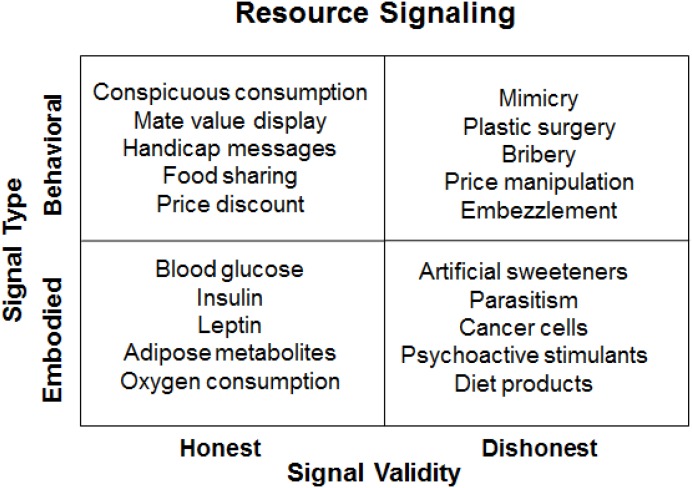
A resource signaling framework.

In a 2 by 2 framework of resource signaling, the types of signals are classified as either behavioral or embodied while the validity of the signaling is assessed as being either honest or dishonest. As illustrated in **Figure 1**, conspicuous consumption is an honest behavioral signal of monetary resource abundance; blood glucose levels are viewed as an honest embodied signal of bodily energy budget; mimicry (e.g., cuckoo strategy) is a dishonest behavioral signal for resource free-riding; artificial sweeteners are a dishonest embodied signal that are used in hope to reduce food intake by making low-calorie food more palatable. In contrast to honest signals, dishonest signals represent an evolutionary mismatch, which occurs when an evolved mechanism is erroneously activated by new stimuli that resemble the original inputs (cues or signals). When the input environment has changed faster than the evolved mechanism can adapt to, evolutionary mismatch may take place, resulting typically in decreased fitness outcomes (see Li et al., 2018).

Honest signaling effectively reduces information asymmetry between a sender and a receiver (Spence, 2002). I argue that the main function of resource signaling is to reduce uncertainty about energy budget and update and forecast trends in resource availability. Thus, honest embodied signals are proactive; and are used in resource management, including caloric metabolism, food intake, feeding, foraging, and other behaviors of resource regulation, such as, self-control, intertemporal choice between an immediate smaller reward and a delayed larger reward, intention for cooperation, etc. Embodied signals, as well as many behavioral signals are sent unintentionally (Spence, 2002). However, these unintentional signals affect intentional decision making. It is possible that blood glucose was not evolved in the first place as a signal but was selected as the primary energy substrate of the brain metabolism. However, once the evolved resource-regulation mechanisms can reliably detect and use blood glucose levels as a proactive indicator (cue) of energy budget, it is then likely to be treated by these mechanisms as a specific signal for resource regulation. From this perspective, blood glucose, given its advantages over other candidates (see more detailed analysis in the next section), can be viewed as a signal evolved from a cue.

## Blood Glucose as a Better Forecasting Signal for Behavioral Management of Resources

Commonly assumed in signaling theories, signals are emitted from a sender to a receiver via a transmission channel. Signaling theory provides a useful framework for a large body of studies in multiple disciplines ranging from anthropology, communication, management science to studies of animal behavior (Shannon and Weaver, 1949; Bird and Smith, 2005; Connelly et al., 2011). However, in embodied metabolic signaling, signals are generated within an individual, and the sender and the receiver of signals are the same organism.

Berthoud (2011) shows that cognition and emotions are in part modulated by signals of nutrient availability. The “cognitive and emotional brain” exerts top-down homeostatic regulation via the cortico-limbic system while the “metabolic brain”, consisting of the hypothalamic and midbrain dopamine pathways receives bottom-up circulating signals of energy availability (e.g., glucose, leptin, and ghrelin) and regulates foraging and feeding behaviors.

There is a group of candidate signals that can provide the brain with valid and prompt caloric information. The following is a short list of embodied signals that are themselves integral parts of the body caloric metabolism: circulating blood glucose, insulin, leptin, oxygen, basal metabolic rate, gastric hormones, ATP molecules, and adipocytes (see Fruhbeck et al., 2001, for a review on the role of the adipocyte in body energy regulation).

Among these candidate signals, which one would most likely be chosen by natural selection as a primary signal regulating both caloric metabolism and behavioral resource management? Is there an embodied signal that can affect both physiological and behavioral aspects of resource management all by itself?

**Table 1** shows candidate metabolic signals and their functional properties. Overall, circulating glucose appears to be a better signal for regulating energy metabolism and behavioral management of resources. The validity, accuracy, and speed of signaling would peak if a caloric signal is also consumed by the receiver for its caloric provision. From this perspective, circulating glucose is a better signal since it is also the only fuel for brain metabolism (Sokoloff, 1989; Falkowska et al., 2015).

**Table 1:** Comparisons between candidates of caloric signals for physiological and behavioral management of resources.

Functional Properties				Candidate caloric signals
	
	Glucose	Insulin	Oxygen	Ghrelin	Leptin	Cortisol	Adipocyte
Brain fuel	Yes	No	Combustion of glucose in aerobic respiration	No	No	No	No (Cannot freely pass the blood brain barrier)
Peripheral fuel	Yes	No	Aerobic respiration	No	No	No	Yes
Receptors	Specific brain receptors	Specific brain receptors	No	Specific brain receptors	Specific brain receptors	Present in almost every cell in the body	Mainly in the gastrointestinal system
Calorie donor	Yes	No	No	No	No	No	No
Channel of signaling	Blood circulation	Blood circulation	Blood circulation	Blood circulation	Blood circulation	Blood circulation	Blood circulation (limited)
Rhythm of effects	Circadian and diet dependent	Circadian and glucose dependent	Respiration of 12–18 breaths per minute in adults	Circadian and diet dependent	Circadian and diet dependent	Stress dependent	Seasonal and stress dependent
Functions in energy balance	Energy conservation and expenditure	Energy conservation (anabolism and glycogenesis)	Energy conservation (aerobic respiration and glycolysis)	Energy consumption (a hunger hormone)	Energy expenditure (a satiety hormone)	Energy expenditure (hyperglycemia and glycogenolysis)	Energy conservation and expenditure
Present in diet	Yes	No	No	No	No	No	Yes
Sensory feedback	Taste of Sweetness	No	No	No	No	No	Possible taste of fat (oleogustus)


In their classic work on communication, Shannon and Weaver (1949) identified for any signaling system the following basic elements: information source, message transmitter, signal transmission channel, signal receiver, and message destination. After an information source produces a message, the message is encoded by a message transmitter into signals which then travel through a channel to be received at the message destination. In the case of resource signaling by circulating glucose, the information source is caloric metabolism; the transmitter is the body cells that consume glucose, the channel is likely the nerves and cardiovascular system; the receiver is the glucose receptors located in the hypothalamus and elsewhere in the brain (Neary et al., 2004); and the signal destination has to be the brain regions regulating metabolic and behavioral resource regulation.

There are multiple advantages of using circulating glucose as a primary signal for behavioral management of resources: (1) A signal has to register in the brain rather than other tissues or organs to have any behavioral effects. Glucose is the primary energy substrate used by adult brain cells (Sokoloff, 1989; Falkowska et al., 2015). For an average person in a fasted and sedentary condition, about 60 percent of blood glucose is consumed by the brain (Wasserman, 2009). Unlike oxygen and ATP molecules used by almost all types of cells in the body, the brain cells selectively consume glucose to sustain their neural activity. (2) Glucose freely passes the blood brain barrier and has specialized receptors in the brain (Neary et al., 2004). (3) Together with insulin, glucagon, and leptin, circulating glucose levels regulate immediate, short-term and long-term energy expenditure and conservation (Havel, 2001). (4) Glucose is naturally present in the human diet. Although there are mutual effects between glucose and insulin, glucagon, oxygen etc., glucose is more directly connected to eating, and triggers insulin reactions but not vice versa in most normal conditions. (5) Humans have an evolved sensory system to detect glucose. The preference for sweetness guides human foraging, feeding, and food choice (Rozin, 1982; Booth, 1994).

Overall, circulating glucose is arguably a better candidate for resource signaling (e.g., being directly related to foraging and eating) and behavioral regulation (e.g., being the selected caloric provider to the brain cells). In the next sections of the paper, I present evidence that circulating glucose levels indeed regulate human decision making in resource management.

## Dual Functions of Glucose: Cellular Fuel and Behavioral Signal

Briers et al. (2006) argue that “people’s desire for money is a modern derivate of their desire for food” (p. 939). From this perspective, money and food are two exchangeable forms of resource, and thus bodily caloric needs would also affect monetary decisions. Consistent with this argument, Briers et al. (2006) found that the participants’ preference to donate to charity decreased when they were hungry. The desire for caloric resources and the desire for monetary resources were positively correlated. Other studies suggest that helping behaviors, as a way of providing resources, are also sensitive to blood glucose levels (see Gailliot and Baumeister, 2007; DeWall et al., 2008). Low blood glucose levels indicate more urgent need for self-preservation than reciprocal future returns.

Wang and Dvorak (2010) have provided more direct evidence of the signaling effects of blood glucose in regulating delay discounting, which refers to the observation that the subjective value of a reward decreases when it is delayed. The results of the study showed that a sugar drink increased blood glucose levels and reduced delay discounting of the participants, as indicated by their greater preference for delayed larger rewards than immediate smaller rewards. In contrast, a diet drink, without a calorie boost, increased delay discounting with a greater preference for immediate smaller rewards than delayed larger rewards, suggesting that fake signals of artificial sweeteners can be detected by the body and thus increase the desire for immediate resource acquisition. It should be noted that energy budget signaling is only sensitive to changes in blood glucose levels within a normal range. In the case of diabetes, blood glucose levels are out of order. Abnormally high levels of blood glucose indicate a caloric crisis of cellular starvation.

**FIGURE 2:**
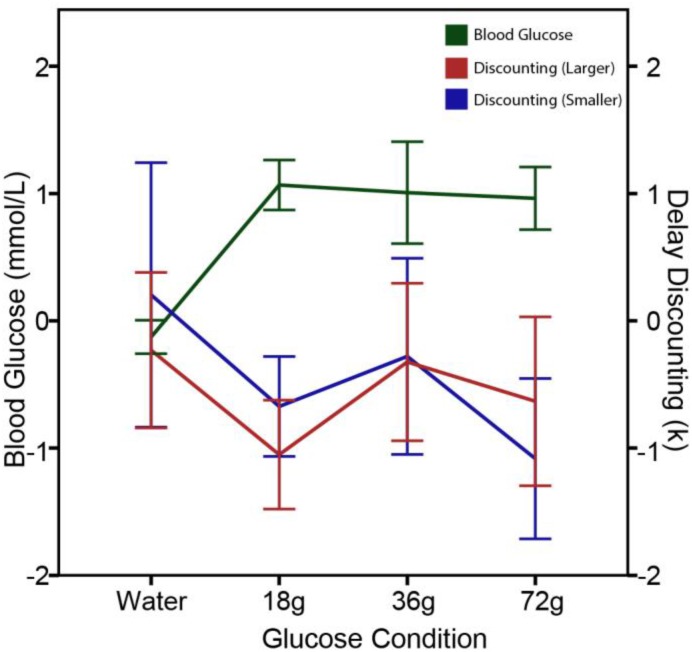
Reduced delay-discounting rate and increased blood glucose levels following glucose administration in Study 1. The figure displays mean changes in blood glucose levels (mmol/L) and in delay-discounting *k* (natural log transformed) 15 min after administered 250 ml water, 18 g sugar drink, 36 g sugar drink, or 72 g sugar drink. A significant decrease in discounting rate after a sugar drink was observed for both the larger ($500–170,000) and smaller ($5–30) rewards. Error bars represent ± 1 standard error of the mean.

To further explore the effects of circulating glucose on delay discounting, Wang and Huangfu (2017) conducted two follow-up studies with additional experimental controls. In Study 1, delay discounting was measured in five conditions: zero consumption, water consumption, and three glucose consumption groups in which fasting participants were administered 18, 36, and 72 g cane sugar in a 250 ml drink, respectively. Intertemporal choice questions with varying delay discounting rates were given before and after a beverage intervention. As shown in **Figure 2**, the results revealed that the rate of delay discounting and the levels of blood glucose were negatively correlated with each other. In all three sugar dink conditions, delay discounting rate was significantly reduced after the sugar administration. In contrast, delay discounting remained unchanged in the two control groups (i.e., the zero consumption and water consumption groups). The lack of significant changes in delay discounting in water consumption group suggests that the effects of circulating glucose on delay discounting are a result of hunger-reduction rather than thirst-reduction. Thus, blood glucose regulates delay discounting via a hunger reduction mechanism.

Study 2 examined input specificity of glucose signaling by testing whether the body can distinguish glucose from other forms of sugar. The result demonstrated that only glucose administration, but not the administration of water or another form of sugar (xylitol) reduced delay discounting and increased future-oriented choices. This result indicates that glucose is likely a specific messenger for signaling changes in body energy budget. Circulating glucose may be a privileged signal favored by natural selection for regulating decisions related to resource management (see **Table 1** for comparative advantages of circulating glucose as an index of body energy condition). Overall, these results reveal a forecasting mechanism carried out by the glucose-insulin system to regulate both metabolic and behavioral acquisition and allocation of resources.

The finding that the behavioral effects of blood glucose levels on delay discounting is directional (increasing or decreasing) but not linearly dose-dependent suggests that the signaling effects of circulating glucose is independent of the amount of calorie provision. In Experiment 1 of the Wang and Huangfu (2017) study, the three doses of sugar drink equally increased the blood glucose levels, showing a celling effect when the sugar dose increased from 18 to 36 and 72 g. The delay discounting rates in these three conditions equally decreased after a sugar drink, closely and reversely followed the changes in blood glucose levels, thus showing a floor effect (see **Figure 2**). It is not the absolute levels but the changes in glucose levels following glucose ingestion or restriction that result in behavioral adjustments. For the people living in modern societies without risk of starvation, energy availability and caloric conditions of the body play more of a role in regulating behaviors of resource management beyond eating and feeding. From this perspective of behavioral regulation, money and glucose are both currencies of resource: one for financial resource and one for caloric resource.

The central view of the resource signaling hypothesis holds that blood glucose fulfills a dual function as the fuel and an anticipatory signal for brain activities. Circulating glucose not only provides calories to the brain but also informs the brain any trendy changes in the body’s energy budget, allowing preemptive behavioral resource regulation. Supporting this viewpoint, a study of foraging bumblebees showed that a small drop of sugar solution, that was insufficient for body energy elevation, can still facilitate foraging choices made by the bumblebees (e.g., Perry et al., 2016). In human studies, manipulations of blood glucose levels affected resource-related decision making, such as political attitudes toward welfare policies (Aarøe and Petersen, 2013), and sensitivity to food and mating cues (Rao et al., 2015).

Based on 42 studies, a meta-analysis by Orquin and Kurzban (2016) examined the effects of blood glucose on four aspects of human decision making: delay discounting, willingness to pay, willingness to work, and decision style. The results show that low levels of blood glucose *increase* desire for resource acquisition, as indicated by higher willingness to pay and willingness to work to get food but *decrease* the desire for monetary spending, as indicated by lower willingness to pay and work for monetary rewards. Low levels of blood glucose made decision makers more impatient. In addition, for monetary rewards only, there was a tendency to make more intuitive than deliberate decisions when blood glucose levels were lower, showing that the effects of visceral cues are reward-type specific. These results are more consistent with a signaling view of glucose as “an input that guides adaptive behavior” (Orquin and Kurzban, 2016, p. 559).

## Addressing the Blood Glucose Debate: Limited Resource, Motivational, and Signaling Accounts

Different views on how circulating glucose affects self-control have been proposed. The limited resource model assumes that self-control consumes glucose and makes glucose supply to the brain a limiting factor for exercising and sustaining self-control against temptations, distractions, and fatigue to complete a task in hand. Thus, exercising self-control may deplete the limited mental resources, particularly when blood glucose levels are low (Baumeister et al., 1998, 2007; Vohs et al., 2008; Baumeister, 2014). From this perspective, self-control is analogous to skeletal muscle in that both experience fatigue. Due to this analogy, the limited resource model is also called the *strength model*. Glucose intake thus should improve the strength of self-control by replenishing resource supply to the brain.

This key prediction of the limited resource model has received support from a series of initial empirical tests. Glucose, but not non-caloric sweeteners, offsets the ego-depletion effect, which refers to a condition where self-control is impaired due to depleted resource for mental activities (Gailliot and Baumeister, 2007; Masicampo and Baumeister, 2008). A meta-analysis of 83 studies (Hagger et al., 2010) confirmed that ego depletion procedures significantly reduce the performance of the participants in self-control tasks, as measured by their effort, reported fatigue, perceived difficulty, and blood glucose levels. Experimental glucose administration improved the performance on a subsequent self-control task. Besides self-control, blood glucose supply has been shown to facilitate a variety of cognitive functions (Holmes et al., 1983; Scholey et al., 2001; Benton, 2010).

However, the limited resource view has been called into question by some researchers holding a motivational view of blood glucose effects. Some more recent studies found little evidence of the anti-ego-depletion effects of glucose (e.g., Lange et al., 2014; Dang, 2016; Vadillo et al., 2016). Other researchers, assuming the existence of a self-control boosting effect of blood glucose, have provided alternative accounts, focusing on either the motivational functions of blood glucose (Molden et al., 2012) or effective allocation mechanisms of the brain to maintain sufficient energy supply to ongoing mental activities (Kurzban, 2010; Beedie and Lane, 2012). From a motivational perspective, glucose supply does not limit mental performance; it instead elevates motivational levels of self-control (Beedie and Lane, 2012, and Carter and McCullough, 2013). Empirical support to this argument comes from a group of studies showing that glucose taste alone is sufficient to offset ego depletion effects (Molden et al., 2012; Sanders et al., 2012; Hagger and Chatzisarantis, 2013). It appears that glucose mouth-rinse without ingestion regulates self-control through motivational instead of caloric mechanisms.

A third view on the behavioral effects of circulating glucose is our signaling hypothesis (Wang and Dvorak, 2010; Wang and Huangfu, 2017). This view of circulating glucose as a signal of resource forecasting emphasizes the anticipatory functions of glucose signaling above and beyond its caloric functions. A minor change in blood glucose levels may be negligible in the context of general metabolism of the brain. However, the same change as a designed signal, may revise the entire energy budget of an organism. The dual functions of calorie provision and energy budget forecasting of circulating glucose would strengthen mental activities in reaction to instant cognitive demands, as the limited resource model would predict, and regulate self-control in anticipation of the resource needs of the body, as the motivational account would predict. Foraging and feeding studies have shown that the sensation of sweetness of glucose is innately pleasant and motivating (Rozin, 1982) while the ingestion of carbohydrates induces satisfaction and satiety (Booth, 1994; Berthoud, 2011).

## Dual Signaling Effects of Glucose Taste and Ingestion

Recently, we conducted a critical test of the signaling effects of glucose by dissociating the effects of glucose taste on self-control from the effects of glucose ingestion on delay discounting (Wang et al., 2018). Moreover, glucose taste and ingestion regulate eating behavior via distinct brain mechanisms (Smeets et al., 2011).

From a dual signaling perspective, for a forager tasting a food sample, the sweet taste of glucose (not non-caloric sweeteners) indicates opportunities of acquiring more caloric and nutritious food. Thus, glucose taste is likely to motivate an individual to keep performing the current foraging task and being present-orientated. In contrast, ingested glucose would indicate that the body has accumulated caloric resources and should be more affordable for future-oriented actions.

To test these predictions, we gave fasted participants a delay-discounting task and a grip-control (self-control) task. The participants performed each task twice, before and after drinking a sugar or non-caloric beverage. The results showed that after a glucose beverage was ingested, delay discounting rate was significantly reduced. The participants became more future-oriented. In contrast, glucose taste without ingestion made the participants more likely to choose smaller-sooner rewards than larger-later rewards, and thus increased their delay discounting. The performance of the participants in the grip-control task was significantly improved by glucose ingestion, but not glucose rinsing. This result suggests that unlike intertemporal choice preference, the physical strength needed in the grip-control task can only be sustained by actual glucose supply.

To further examine the neural basis of glucose dual signaling, an fMRI study was conducted. We first identified from the literature a number of brain structures implicated in delay discounting, including the *anterior cingulate gyrus* (Monterosso et al., 2007; Pine et al., 2009), the *medial prefrontal cortex* (Kable and Glimcher, 2007; Marco-Pallarés et al., 2010), the *middle and inferior frontal gyri* (Kishinevsky et al., 2012), and the *posterior cingulate cortex* (McClure et al., 2004; Kable and Glimcher, 2007). These particular structures show greater activation when the subjective values of the immediate and delayed options are similar, causing the choice more difficult to make. Lower activation in these structures is associated with greater impulsivity and a higher rate of weight gain over a period of 1.3–2.9 years after the participation in the study, possibly due to enhanced impulsivity (e.g., Kishinevsky et al., 2012). We predict that glucose taste, compared to glucose ingestion, is more likely to increase the activities in these delay-discounting and self-control related brain regions.

Seventeen fasted healthy participants completed three sets of intertemporal choice (delay discounting) tests while an fMRI scan was performed. The participants started with a baseline delay discounting test following by a second test after glucose mouth rinse, and then a final delay discounting test after glucose ingestion. There was a 15 min of break between the second test and the final test. In this within-subject experiment, the order of the tasting and ingestion was consistent with the natural order of tasting food in the mouth before ingestion.

The brain-activation pattern after glucose rinse was distinct from the pattern observed following glucose ingestion. Glucose rinsing had greater activation than glucose ingestion in several brain regions, including the *anterior* *cingulate gyrus*, the *inferior frontal gyrus*, and the *superior temporal gyrus*. These three cortical structures were found to be more active when dealing with more difficult intertemporal choices (e.g., Monterosso et al., 2007; Kishinevsky et al., 2012). Together, these results suggest that actual ingestion of glucose can reduce ambiguity in choice preference and thus facilitate the process of intertemporal decision making.

In sum, our findings reveal a dual signaling role of glucose sensation and ingestion in regulating time-preference and self-control. Sweet taste, without actual caloric supply, signals opportunities of resource acquisition and thus motivates resource acquisition and present orientation. In contrast, actual ingestion, similar to money in hand, indicates resource abundance and thus increases future orientation and even reduces the motivation. Fung et al. (2017) found that participants were more likely to quit in a task of persistence either when the rate of monetary rewards was higher or after consumed a caloric drink. Gaining both types of resource (caloric drink and money) reduced the motivation of the participants for further resource acquisition.

## Fake Resource Signaling and Cheating Detection in Energy Metabolism

As illustrated in **Figure 1**, embodied resource signaling can be either genuine or fake. Can our body distinguish real glucose from artificial sweeteners? Would the body detect artificial sweeteners as fake signals of caloric supply and react with countermeasures? Accumulating evidence suggests that the body after prolonged experience with artificial sweeteners can detect the fake signals, and in return increase caloric intake (e.g.,Stellman and Garfinkel, 1986; Swithers and Davidson, 2008; Hill et al., 2014) and become more present-oriented (Wang and Dvorak, 2010).

Hill et al. (2014) tested whether consumption of artificial sweeteners affects individuals’ evaluation and choice of food. In three experiments, the authors found that consuming a non-caloric sweetened beverage, compared to both a sugar-sweetened and an unsweetened beverage, increased the salience of high-calorie foods, increased the likelihood that participants would choose a high-calorie snack when making a hypothetical consumer choice, and decreased how satisfied participants felt after consuming a sugar-sweetened snack. These results suggest that the body can detect non-caloric sweeteners as fake signals in body energy metabolism, and in reaction, increase calorie intake.

Long-term use of artificial sweeteners can increase body weight and obesity (Stellman and Garfinkel, 1986; Swithers and Davidson, 2008). This finding suggests that the body is able to detect artificial sweeteners as a signal of caloric crisis, and thus increases caloric intake in return. One of the behavioral changes due to chronic use of artificial sweeteners is a stronger hedonic response to tempting food. Dieters showed stronger favorable reactions to the tempting-food stimuli than normal controls (Hofmann et al., 2010).

Further evidence shows that habitual consumption of artificial sweetener (i.e., diet soda) interferes with the reward processing of sweet taste in the brain. Green and Murphy (2012) examined the fMRI response of 12 diet drinkers and 12 non-diet drinkers to sucrose (a caloric sweetener) and saccharin (a non-caloric sweetener) while performing hedonic evaluations of the drink. Regular diet soda drinkers (1.14 diet sodas per day, on average) displayed greater activation to both drinks in the right amygdala and the ventral tegmental area of the dopaminergic reward pathway. For people who did not consume diet sodas, saccharin resulted in greater responses than sucrose in the right orbitofrontal cortex. In addition, heavier diet drinkers had reduced activation in the caudate nucleus. These results suggest that habitual drinking of diet soda can result in alterations in the brain regions implicated in the processing of the taste, smell, and sight of food reward.

In a pilot study, we found that compared to non-diet drinkers, diet drinkers were more impulsive as indicated by their higher delay discounting rate and greater intention to spend than save money. These differences are likely a result of diet experience since the delay discounting rate of the participants increased with their average weekly consumption of diet drink. In contrast, the average weekly consumption of sugary drink had no effect on the delay discounting rate of the participants.

Artificial sweeteners break the evolutionary link between sweet taste and its calorie provision function. One of the brain structures that may be involved in detecting such false signaling is the amygdala. With an initial dose, the amygdala shows a greater activation to non-caloric than caloric beverage (Smeets et al., 2011). Over time, the use of the artificial sweeteners reduced the amygdala response to sugar consumption (Rudenga and Small, 2012).

## Conclusion

Compared to the motivational and limited-resource models, our hypothesis of resource signaling via blood glucose is able to account for both the motivational and the resource-provision functions of blood glucose. Blood glucose as the sole carrier of caloric resource to brain cells also provides the brain with information about ups and downs in body energy budget. Thus, small changes in blood glucose levels as signals of energy budget can proactively trigger significant metabolic and behavioral reactions. In his seminal book, James(1892, p. 222) provided a powerful example of magnifying effects of signaling: “A faint tap *per se* is not an interesting sound; it may well escape being discriminated from the general rumor of the world. But when it is a signal, as that of a lover on the window-pane, hardly will it go unperceived.” Similarly, minute changes in circulating blood glucose forecast body energy budget and regulate intertemporal choice. A declining trend in blood glucose levels and in energy budget should increase the preference for present rewards to avoid possible harms to survival while an improving trend in blood glucose levels and in energy budget should promote the preference for future rewards to maximize reproductive potentials.

The dual functions (caloric provision and resource forecasting) and dual signaling (glucose taste and ingestion) hypothesis of behavioral effects of circulating glucose reconciles some key differences between the motivational views (Molden et al., 2012) and the limited resource view (Gailliot and Baumeister, 2007). The motivational views are correct in suggesting that the motivating sweet taste of glucose shifts attention to present rewards while the limited resource model is correct in predicting that glucose supply improves future orientation and self-control (Wang et al., 2018).

Accumulating evidence suggests that visceral signals of circulating glucose play an important role in rational decision making and self-control. From this perspective, some cognitive anomalies in decision making may be better viewed as adaptations to energy conditions of the body. For instance, a typical phenomenon of inconsistent delay discounting identified in the literature of behavioral economics is the absolute magnitude effect where people discount smaller rewards more than larger rewards (Loewenstein and Prelec, 1992). Ballard et al. (2017) argue that this magnitude effect can be viewed as a reasonable strengthening in self-control for bigger rewards. In fact, high-magnitude rewards augment activation levels of the frontal executive-control regions of the brain. Moreover, the magnitude effect is sensitive to the caloric condition of the body. Increased hunger reduced the magnitude effect, suggesting a down-regulation of future orientation by visceral cues of greater needs for immediate resource acquisition.

If glucose is a genuine signal of energy metabolism of the body, non-caloric sweeteners can be viewed as signals of “metabolic cheating”. Body adapts over time to such dishonest signals with countermeasures, including increased resource acquisition, reduced future orientation, and distinct neurophysiological changes. As a result, habitual dieters show greater delay discounting and impulsivity. These findings are alarming given the popularity of non-caloric sweeteners among consumers as a means of endorsing a healthy body weight and healthy life style. In the United States, about 25 percent of children and 41 percent of adults reported that they consumed foods and beverages containing artificial sweeteners. From 1999 to 2012, the users of artificial sweeteners in the United States grew by 54 percent for adults, and were tripled for children (George Washington University Milken Institute School of Public Health, 2017). Taken together with previous findings (e.g., Wang et al., 2018), impulsive behaviors may be aggravated by either the chronic consumption of non-caloric sweeteners or appetite-inducing stimulations without timely caloric provision. Avoiding sharp fluctuations in blood glucose levels may be a useful means of reducing impulsive behaviors and promoting future orientation.

## Ethics Statement

The studies conducted by the author were carried out in accordance with the recommendations of American Psychological Association, and were approved by the Institutional Review Board of the University of South Dakota. All subjects gave written informed consent for their participation.

## Author Contributions

The author confirms being the sole contributor of this work and has approved it for publication.

## Conflict of Interest Statement

The author declares that the research was conducted in the absence of any commercial or financial relationships that could be construed as a potential conflict of interest.
